# Fecal Lcn-2 level is a sensitive biological indicator for gut dysbiosis and intestinal inflammation in multiple sclerosis

**DOI:** 10.3389/fimmu.2022.1015372

**Published:** 2022-10-21

**Authors:** Sudhir K. Yadav, Naoko Ito, John E. Mindur, Hetal Kumar, Mysra Youssef, Shradha Suresh, Ratuja Kulkarni, Yaritza Rosario, Konstantin E. Balashov, Suhayl Dhib-Jalbut, Kouichi Ito

**Affiliations:** ^1^ Department of Neurology, Rutgers-Robert Wood Johnson Medical School, Piscataway, NJ, United States; ^2^ Department of Clinical and Chemical Pathology, National Research Centre, Dokki, Egypt; ^3^ Department of Neurology, Rutgers-New Jersey Medical School, Newark, NJ, United States

**Keywords:** multiple sclerosis, gut dysbiosis, colitis, neutrophils, lipocalin-2 (LCN-2), experimental autoimmune encephalomyelitis (EAE), biomarker

## Abstract

Multiple Sclerosis (MS) has been reported to be associated with intestinal inflammation and gut dysbiosis. To elucidate the underlying biology of MS-linked gut inflammation, we investigated gut infiltration of immune cells during the development of spontaneous experimental autoimmune encephalomyelitis (EAE) in humanized transgenic (Tg) mice expressing HLA-DR2a and human T cell receptor (TCR) specific for myelin basic protein peptide (MBP87-99)/HLA-DR2a complexes. Strikingly, we noted the simultaneous development of EAE and colitis, suggesting a link between autoimmune diseases of the central nervous system (CNS) and intestinal inflammation. Examination of the colon in these mice revealed the infiltration of MBP-specific Th17 cells as well as recruitment of neutrophils. Furthermore, we observed that fecal Lipocalin-2 (Lcn-2), a biomarker of intestinal inflammation, was significantly elevated and predominantly produced by the gut-infiltrating neutrophils. We then extended our findings to MS patients and demonstrate that their fecal Lcn-2 levels are significantly elevated compared to healthy donors (HDs). The elevation of fecal Lcn-2 levels correlated with reduced bacterial diversity and increased levels of other intestinal inflammation markers including neutrophil elastase and calprotectin. Of interest, bacteria thought to be beneficial for inflammatory bowel disease (IBD) such as *Anaerobutyricum, Blautia, and Roseburia*, were reduced in fecal Lcn-2-high MS patients. We also observed a decreasing trend in serum acetate (a short-chain fatty acid) levels in MS Lcn-2-high patients compared to HDs. Furthermore, a decrease in the relative abundance of *Blautia massiliensis* was significantly associated with a reduction of acetate in the serum of MS patients. This study suggests that gut infiltration of Th17 cells and recruitment of neutrophils are associated with the development of gut dysbiosis and intestinal inflammation, and that fecal Lcn-2 level is a sensitive biological indicator for gut dysbiosis in multiple sclerosis.

## Introduction

Multiple sclerosis (MS) is an immune-mediated neuroinflammatory disease that causes demyelination and axonal loss in the central nervous system (CNS) (1). Recent studies suggest that gut dysbiosis is a risk factor for disease progression (2, 3). For example, a significant decrease in Clostridium clusters XIVa and IV was observed in patients with RRMS (4). Clostridium *clusters* have the potential to induce Foxp3^+^ T regulatory cells (Tregs), which suppress inflammatory conditions, by metabolizing fiber into short-chain fatty acids (SCFAs) (5–7). Therefore, decreases in the abundance of Clostridium *clusters* may potentially increase susceptibility to relapsing-remitting multiple sclerosis (RRMS) (4). The abundance of other SCFA-producing gut bacteria including *Butyricimonas* and Prevotella *which promote Foxp3+ Treg development*, are also decreased in RRMS patients (8). In contrast, mucin-degrading bacteria *Akkermansia Muciniphila*, Th17-inducing bacteria Streptococcus mitis, and *Methanobrevibacter* are reportedly increased in RRMS patients compared to healthy donors (HDs) (8–10). These studies suggest that gut microbiome imbalance between anti-inflammatory and pro-inflammatory bacteria may increase the risk for autoimmune diseases including MS.

The homeostasis of gut bacteria is regulated in part by the immune system. Under steady state conditions, an excessive activation of immune cells against gut bacteria is suppressed by regulatory immune cells induced by microbial-derived signals (11). However, microbial homeostasis in the alimentary tract can sometimes break under the influence of environmental factors, including diet, infection, antibiotics, and stress, leading to the onset of gut dysbiosis (12). When pathobionts expand in the setting of gut dysbiosis, innate and adaptive immune cells become primed to eliminate them. Neutrophils are a critically important cell subset for responding to pathobionts that favor gut dysbiosis. These innate leukocytes are recruited to the gut in an IL-17A- and CXCR2-dependent manner in response to the IL-17A produced by activated innate and adaptive immune cells in the gut mucosa (11, 13). NOD2 signaling induced by pathobionts also recruits neutrophils (14). The recruited neutrophils eradicate pathobionts by producing antimicrobial proteins, such as elastase and reactive oxygen species (ROS) (15, 16). Neutrophils that have infiltrated the gut mucosa also produce lipocalin-2 (Lcn-2)/neutrophil gelatinase associated lipocalin (NGAL), an innate immune factor that suppresses the proliferation of pathobionts by interacting with bacterial siderophores to limit iron acquisition and subsequent bacterial growth (17, 18). Lcn-2 also enhances phagocytic bacterial clearance in macrophages and promotes antimicrobial activity by preserving myeloperoxidase activity in neutrophils (19, 20). Therefore, Lcn-2 produced by infiltrated neutrophils serves as a biological indicator of gut dysbiosis. In addition, pro-inflammatory cytokines including IFN-γ, TNF-α, IL-17A, IL-6, and IL-1β, which are secreted during gut inflammation, can augment the production of Lcn-2 in an NF-κB-dependent manner in epithelial cells, macrophages, and infiltrated neutrophils (21). Thus, fecal Lcn-2 is a secreted innate factor that appears to serve as both a biomarker of gut dysbiosis and intestinal inflammation (22–24).

While the activation of neutrophils is critical for eliminating expanding pathobionts, neutrophil activation can also function as a double-edged sword by inducing tissue damage in the gut. Activated neutrophils release oxidants and proteases, such as matrix metalloproteases and elastase, that can cause tissue injury (25). Inflammatory bowel disease (IBD) is thought to be triggered by the activation of intestinal immune cells against gut microbes. A hallmark sign of active IBD is neutrophil infiltration in intestinal tissues, although whether infiltrated neutrophils play a harmful or protective role is controversial (26, 27). Of note, MS is reportedly associated with IBD based on epidemiological studies which highlight their reciprocal comorbidity (28–33). Moreover, shared genetic risk factors between MS and IBD have also been described (34). Likewise, recent murine studies revealed that Th17 cells infiltrate the gut during the development of EAE, and that stem-like intestinal Th17 cells, which are maintained by gut microbes, can give rise to pathogenic effector T cells that traffic to the CNS (35–37). The enrichment of Th17 cells in the gut was also previously reported during MS progression (2). Therefore, we hypothesized that IL-17A produced in the gut prior to or during the onset of CNS autoimmunity mediates the infiltration of neutrophils, which contribute to MS-associated gut dysbiosis and intestinal inflammation. To test this hypothesis, we assessed whether the infiltration of neutrophils and production of Lcn-2 are linked to EAE- and MS-associated gut dysbiosis, intestinal inflammation, and alteration in short-chain fatty acids.

## Materials and methods

### Animals

All experiments were carried out in compliance with the Rutgers Institutional Animal Care and Use Committee guidelines (Protocol number: 999900130). 3A6/DR2a Tg mice were created using a 3A6 T cell clone isolated from an MS patient (38). The development of spontaneous EAE was monitored from the age of 3-weeks-old until 30-weeks-old. Clinical scores were measured as follows: 0: no signs of disease; 1: limp tail; 1.5: paresis of one hindlimb; 2: paresis of both hindlimbs; 2.5: paralysis of one hindlimb; 3.0: paralysis of both hindlimbs; 3.5: paralysis of both hindlimbs and one forelimb paresis; 4: hindlimb paralysis and both forelimb paresis; 5: no mobility/moribund. Fecal samples were collected by placing individual mouse in an empty container for 1-2 hours, and 5-10 fecal pellets were collected for Lcn-2 ELISA and 16S rRNA sequencing.

### Human subjects

Institutional Review Board approval (Approval number: 20160001256) was obtained from Rutgers-Robert Wood Johnson Medical School (RWJMS), and all subjects were provided written informed consent. RRMS subjects as defined by the 2017 revised McDonald Criteria were recruited from the RWJMS Multiple Sclerosis Center (39). Healthy controls were recruited from local New Jersey residents. All patients and controls were recruited from the same geographic region. All enrollees were between the ages of 18 and 60. Exclusion criteria were: use of any antibiotics within 6 months; use of probiotics within 2 months; inflammatory bowel disease; use of probiotics within 2 months before collection of fecal samples. One participant in each of the MS and HD groups were eating a vegetarian diet; other participants were eating a western diet. Participants were provided with a stool collection kit including an airtight container, commode stool collection container, ice packs, and an anerobic sachet. Once the sample is collected in the stool collection container, it is placed in the airtight container with the anerobic sachet and ice pack. Then, samples were brought into the research laboratory within 24 hours of stool collection. Human fecal samples were aliquoted in 2 mL cryovials in an anaerobic chamber (Sheldon Manufacturing, Cornelius, Oregon) and rapidly frozen using liquid nitrogen. Then, cryovials were kept in -80 °C for long term storage. Demographic information of the study cohort is provided in **Table 1**.

**Table 1:** Demographics of study subjects.

Parameters	Healthy donors (n=18)	MS patients (n=14)	P value
**Age (years)**	36 ± 11.22	43.52 ± 9.61	0.055
**Gender**			0.721
Male (%)	7 (39%)	4 (29%)	
Female (%)	11 (61%)	10 (71%)	
**Race/ethnicity**			>0.999
Caucasian	13	10	
African American	3	3	
Hispanic	2	1	
**MS type**			
RRMS	Not Applicable	14	
PMS	Not Applicable	0	
PMS	Not Applicable	0	
**Disease duration (years)**	Not Applicable	8.6+/-5.7	
**Symptom when fecal samples were collected**			
Flare-up	Not Applicable	0	
Remission	Not Applicable	14	
**Therapy**			
Glatiramer acetate	Not Applicable	4	
Dimethyl fumarate	Not Applicable	5	
Triflunomide	Not Applicable	1	
Interferon beta-1a	Not Applicable	1	
Azilect	Not Applicable	1	
None	Not Applicable	2	

### Isolation of mouse lamina propria cells from intestines

The small and large intestines were flushed with PBS and cut longitudinally along its length before being cut into smaller pieces. After three washes, intestinal pieces were transferred to a 50 mL tube in 30 mL Hank’s balanced salt solution (HBSS) without calcium and magnesium. EDTA (0.5 M) and DTT (1 M) were then added, and the tissue suspension was incubated under gentle shaking at 37°C for 30 min. Epithelial cells and intraepithelial lymphocytes (IEL) were separated by passing supernatant through a 70-micron strainer. The remaining tissue was washed with RPMI media containing 5% FBS and transferred to a new 50 mL tube. 30 mL RPMI media containing 5% FBS was added into the tissue and digested with 100U/mL collagenase IV (Worthington Biochemical Corporation, Lakewood, NJ) and 50 U/mL DNase (Sigma, St.Louis, MO) with shaking at 37°C for 30 min. Lamina propria (LP) cells were isolated from the flowthrough by filtering through a 70-micron strainer, followed by isolation of lamina propria cells using 40% Percoll. Lamina propria cells were obtained as a pellet, which was then washed and suspended in RPMI media containing 10% FBS for further analysis.

### Flow cytometry

Anti-CD4, -CD45, –IFN-γ, and –IL-17 mAbs were all purchased from eBioscience, San Diego, CA. The mAbs against human TCR Vβ5.1 were purchased from Beckman Coulter. Flow cytometry analysis was performed on Gallios and analyzed by Kaluza Software (Beckman Coulter, Brea, CA). Mononuclear cells from the CNS and lamina propria of large and small intestines were stimulated with MBP87-99 (10 μg/mL) and stained with mAbs against CD45, CD4, IFN-γ, IL-17 and human TCR Vβ5.1. CD45^+^ Vβ5.1^+^ cells were gated for analysis of Th1 (CD4^+^ IFN-γ^+^), and Th17 (CD4^+^ IL-17A^+^) cells. To measure the total number of infiltrated cells (CD4^+^ Vβ5.1^+^, CD4^+^ Vβ5.1^+^ IFN-γ^+^, and CD4^+^ Vβ5.1^+^IL-17A^+^) in the CNS, mononuclear cells isolated from the brain and spinal cord were counted and then stained with anti-CD45, CD4, Vβ5.1, IFN-γ, and IL-17 A antibodies. The cell number was calculated based on a percentage of CD45^+^CD4^+^ Vβ5.1^+^, CD45^+^CD4^+^ Vβ5.1^+^ IFN-γ^+^, and CD45^+^CD4^+^ Vβ5.1^+^IL-17A^+^.

For the migration of MBP-specific Tg T cells into the large intestine, spleen cells isolated from spontaneous EAE mice were labeled with CellTrace Violet (Thermofisher, Waltham, MA) and 1x10^7^ cells were injected into healthy 3A6/DR2a Tg mice. Migration of 3A6 TCR Tg T cells (Vβ5.1^+^CD3^+^) into the spleen, MLN (mesenteric lymph nodes), CLN (cervical lymph nodes), CNS (brain and spinal cord), SI (small intestine), and LI (large intestine) was analyzed by gating CellTrace Violet^+^ CD45^+^ cells.

### Immunohistology

Animals were anaesthetized and perfused intracardially with 30 mL ice-cold PBS, followed by 100 mL of 4% paraformaldehyde (PFA). Colon was removed, luminal content was cleaned using PBS, and tissue was kept in 4% paraformaldehyde for 24 hrs. Then, tissue was soaked in 30% sucrose/PBS for 3 days. Cryoblocks of the colon were made by using optimal cutting temperature (O.C.T) (Thermofisher, Waltham, MA) and were cut at a thickness of 10 µm by cryostat and stained with goat anti-Lcn-2 Ab (Abcam, Cambridge, MA) and rat anti-Ly6G Ab (Calbiochem, St. Louis, MO). Donkey anti-goat IgG Rhodamine, and donkey anti-rat IgG Alexa 488 (Jackson Immunoresearch Laboratories, West Grove, PA) were used as secondary antibodies. Cell nuclei were counterstained with DAPI. Digital images of sections were captured by Leica DMi8 fluorescent microscope using LAS X software (Leica, Wetzlar, Germany). Large and small intestines were divided into 4 equal sections and stained. Number of Lcn-2^+^, Ly6G^+^ and Lcn-2^+^Ly6G^+^ double positive cells and tissue area for each section were determined using QuPath software (40). The number of infiltrated cells within the section is presented using number of cells/mm^2^.

### Evaluation of colitis

To evaluate the severity of colitis in 3A6/DR2a mice, H&E staining of colonic sections was prepared at AML Laboratories (Augustine, FL), and colitis severity was scored by Jackson Laboratory pathology service as follows; Normal = 0, Minimal = 1 (generally focal affecting 1-10% of mucosa, or if diffuse then minimal), Mild = 2 (generally focal affecting 11- 25% of mucosa, or if diffuse then mild), Moderate = 3 (26-50% of mucosa affected with areas of gland loss replaced by inflammatory cell infiltrate, milder in remaining areas of mucosa), Marked = 4 (51-75% of mucosa affected with areas of gland loss replaced by inflammatory cell infiltrate, milder in remaining areas of mucosa), Severe = 5 (76-100% of mucosa affected with areas of gland loss replaced by inflammatory cell infiltrate, milder in remaining areas of mucosa) (41).

#### Analysis of cytokine production

Human PBMCs were isolated from whole blood by using lymphocyte separation medium (Thermo Fisher Scientific, Waltham, MA). The freshly isolated PBMCs were cultured at 2 × 10^6^/ml cell density unstimulated or with LPS (*In vivo*Gen, San Diego, CA) at 5 μg/ml, or CD3/CD28 mAbs (Biolegend, San Diego, CA) at 2 µg/ml for 3 days, and cytokine production was examined by ELISA (Biolegend, San Diego, CA). The remaining cells were frozen and stored in liquid nitrogen.

### ELISA assay for Lcn-2, neutrophil elastase, and calprotectin

To measure the levels of Lcn-2, calprotectin, and elastase, fecal samples (~200 mg) were suspended in 0.5 mL of 0.01% Tween 20 in PBS solution, homogenized using sterile disposable pellet pestles (Fisher Scientific, Waltham, MA), and vortexed. The supernatant was collected by centrifugation at 12000 rpm for 10 min, and Lcn-2 concentration in the fecal supernatants was measured by mouse or human Lipocalin-2/NGAL Quantikine kit (R&D systems, Minneapolis, MN). Human fecal calprotectin was measured using an ELISA kit (Raybiotech, Peachtree Corners, GA). Human fecal neutrophil elastase was measured using an ELISA kit (Immundiagnostik AG, Bensheim, Germany). Total protein of fecal supernatant was measured by the Pierce™ BCA Protein Assay Kit (ThermoFisher, Waltham, MA). Lcn-2, calprotectin, and elastase levels were presented by ng per mg of total fecal protein. Serum endotoxin activity was measured using Human endotoxin (ET) ELISA Kit (MyBioSource, San Diego, CA) according to the manufacturer’s recommendation.

### 16S ribosomal RNA sequencing

Fecal DNA was purified by the Fast DNA Stool Mini Kit (QIAGEN, Hilden, Germany) according to the manufacturer’s instructions. The concentration of extracted DNA was determined by Nanodrop 1000 (Thermo Scientific, Waltham MA). For human gut microbiota study, 16S rRNA gene sequencing was done by Zymo Research (Irvine, CA) using custom designed primers to provide the best coverage of the V3-V4 region of 16S rRNA gene while maintaining high sensitivity. The final library was sequenced on Illumina^®^ MiSeq™ with a v3 reagent kit (600 cycles). The sequencing was performed with 10% PhiX spike-in. An average of 33811 sequences per sample were obtained (S.D. ± 8876). Unique amplicon sequence (ASV) variants were inferred from raw reads using the DADA2 pipeline (42). Chimeric sequences were also removed with the DADA2 pipeline. Taxonomy assignment was performed using Uclust from QIIME1 (v.1.9.1) with the Zymo Research Database, a 16S database that is internally designed and curated (43), and any unassigned taxa were further identified by NCBI sequence databases. Alpha and beta-diversity analysis was performed based on rarefied abundance matrix. Taxonomy that has significant abundance among different groups were identified by performing statistical tests on relative abundance across groups.

### SCFAs analysis

Fecal and serum SCFAs were measured by microbiomeinsights (Richmond, BC, Canada) and Creative proteomics (Shirley, NY, USA), respectively.

### Statistical analysis

GraphPad Prism Software version 9 (GraphPad Software, Inc., San Diego, CA) and R version 4.0.2 were used for statistical analyses. Spontaneous EAE clinical scores were evaluated by the non-parametric Mann-Whitney U test. Multiple comparisons were performed with one-way ANOVA with Tukey’s Multiple Comparison Test or Kruskal Wallis test with Dunn’s Multiple Comparison test. Student’s *t* test (unpaired) or Mann-Whitney U test was used to assess the differences between two groups. Fisher exact test was used for categorical variables. The Spearman correlation or Pearson correlation test was used to analyze correlation between parameters. All p-values ≤ 0.05 were considered statistically significant.

## Results

### MBP-specific T cells increase in the large intestine during the development of spontaneous EAE

**Figure 1:**
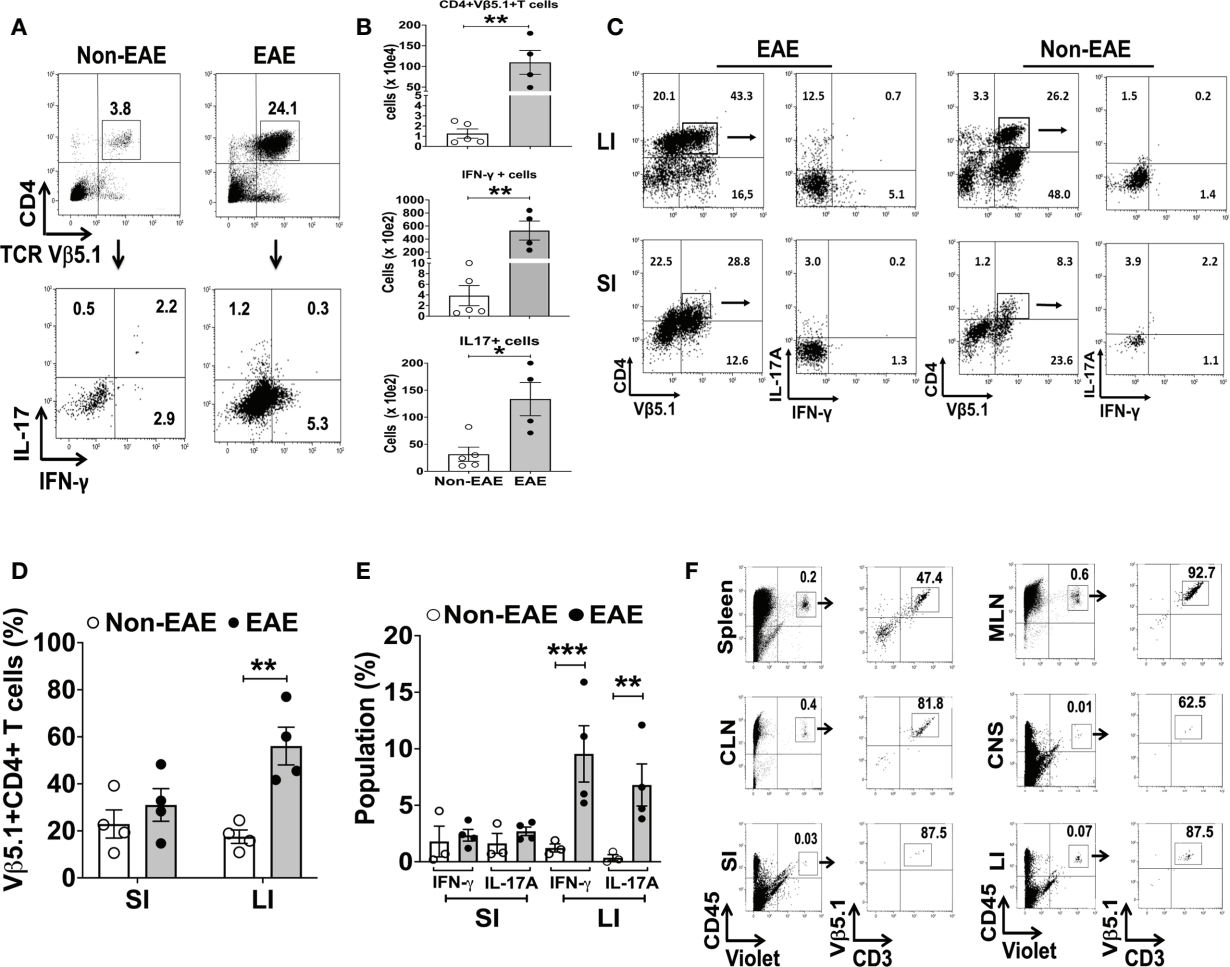
Infiltration of MBP-specific T cells in the large intestine during the development of spontaneous EAE in 3A6/DR2a mice. **(A)** Infiltration of CD4^+^ MBP-TCR (Vβ5.1)^+^ Tg T cells in the CNS and production of IFN-γ and IL-17A in response to MBP87-99 (10 μg/mL). **(B)** Total number of CNS-infiltrated CD4^+^ MBP-TCR (Vβ5.1) Tg T cells, Th1 (CD4^+^ Vβ5.1^+^IFN-γ^+^) cells, and Th17 (CD4^+^ Vβ5.1^+^Th17A^+^) cells (n = 4-5 in each group). **(C, D)** Enrichment of CD4^+^ MBP-TCR (Vβ5.1)^+^ Tg T cells in the large intestine in EAE or non-EAE mice and the percentage of their population within CD45^+^ leukocytes (n = 4 in each group). **(C, E)** Enrichment of MBP-specific Th1 (CD4^+^ IFN-γ^+^) and Th17 (CD4^+^ Th17A^+^) cells in the large and small intestines upon development of EAE and their frequency within CD45^+^ leukocytes (n = 3-4 in each group). **(F)** Migration of MBP-TCR Tg T cells (Vβ5.1^+^CD3^+^) into the large intestine. The spleen cells isolated from spontaneous EAE were labeled with CellTrace Violet and 1x10^7^ cells were injected into healthy 3A6/DR2a Tg mice. Migration of MBP-TCR Tg T cells (Vβ5.1^+^CD3^+^) into the spleen, MLN (mesenteric lymph nodes), CLN (cervical lymph nodes), CNS (brain and spinal cord), small intestine, and large intestine is shown. SI, small intestine; LI, large intestine. Mean ± SEM. *P < 0.05, **P < 0.01, ***P < 0.001.

3A6/DR2a Tg mice harbor T cells expressing the MBP-specific human 3A6 TCR and an MS-linked MHC class II restriction element, HLA-DR2a (38). These mice develop EAE spontaneously during the period of adolescence and young adulthood (44). Since we previously demonstrated that gut dysbiosis was highly associated with spontaneous EAE in 3A6/DR2a Tg animals (44), we analyzed the development and/or infiltration of Th1 and Th17 cells in both the gastrointestinal tract and central nervous system (CNS) in these mice. Expectedly, an enrichment of MBP-specific Th1 and Th17 cells was observed in the CNS upon development of spontaneous EAE (**Figures 1A, B**). Interestingly, MBP-specific Th1 and Th17 cells were also enriched in the large intestines of these mice (**Figures 1C–E**). This enrichment was more significantly pronounced in the large intestine compared to the small intestine. To investigate whether MBP-specific T cells can migrate into the large intestine in 3A6/DR2a Tg mice, splenocytes were isolated from animals with spontaneous EAE, labeled with CellTrace Violet, and injected into naive 3A6/DR2a Tg mice. As shown in **Figure 1F**, MBP-specific CD4^+^ T cell infiltration into the large intestine was detected based on the CellTrace Violet signal. Taken together, our data suggest that the infiltration and/or proliferation of MBP-specific Th1 and Th17 cells in the large intestine increases during the development of spontaneous EAE in 3A6/DR2a Tg mice.

### Intestinal inflammation is associated with the development of EAE

**Figure 2:**
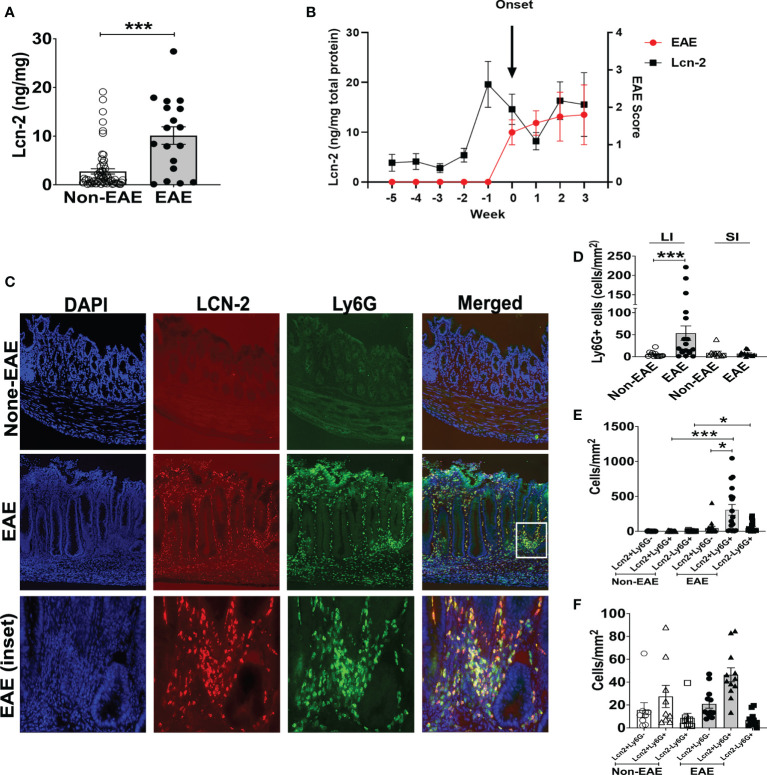
Fecal Lcn-2 is a biomarker for the development of spontaneous EAE in 3A6/DR2a mice. **(A)** Association between increase in fecal Lcn-2 levels (ng/mg total protein) and development of spontaneous EAE (Non-EAE; n = 62, EAE; n = 18).**(B)** Time course of development of spontaneous EAE (•) and fecal Lcn-2 levels (◼) in 3A6/DR2a Tg mice. Fecal Lcn-2 levels before and after clinical EAE onset are shown. Mean ± SEM (n =6). **(C)** Immunohistopathology showing increase in Lcn2^+^ Ly6G^+^ cells in the large intestine upon development of EAE. **(D)** Increase in neutrophil (Ly6G^+^) cells in the large intestine (LI) upon development of EAE. **(E, F)** Large intestines **(E)** and small intestine **(F)** were stained with anti-Lcn-2 mAb and anti-Ly6G mAb, and number of Lcn-2^+^(red), Ly6G^+^ (green), and Lcn-2^+^ Ly6G^+^ (yellow) cells were quantified. Mean ± SEM. *P < 0.05, ***P < 0.001.

The expansion of Th17 cells in the gut is observed during intestinal inflammation (45). Hence, we examined the association between EAE and intestinal inflammation in 3A6/DR2a Tg mice. Since fecal Lcn-2 is a sensitive biomarker of intestinal inflammation (23, 24), we assessed levels of fecal Lcn-2 in EAE and non-EAE mice. Notably, an increase in fecal Lcn-2 levels was significantly associated with the development of spontaneous EAE (**Figure 2A**), and a time course experiment showed that an increase in fecal Lcn-2 levels began one week before symptom onset (**Figure 2B**). Since Lcn-2 is produced by infiltrated Ly6G^+^ neutrophils during gut inflammation (46, 47), we examined the gut infiltration of Ly6G^+^ cells by immunohistology. Of interest, a massive infiltration of Ly6G^+^ neutrophils were observed in the large intestine of EAE mice (**Figures 2C, D**), and Lcn-2 was mainly produced by Ly6G^+^ neutrophils (**Figures 2C, E, F**). Together, these data indicate that Lcn-2^+^ neutrophils localize in the large intestines of 3A6/DR2a Tg mice that develop spontaneous EAE.

**Figure 3:**
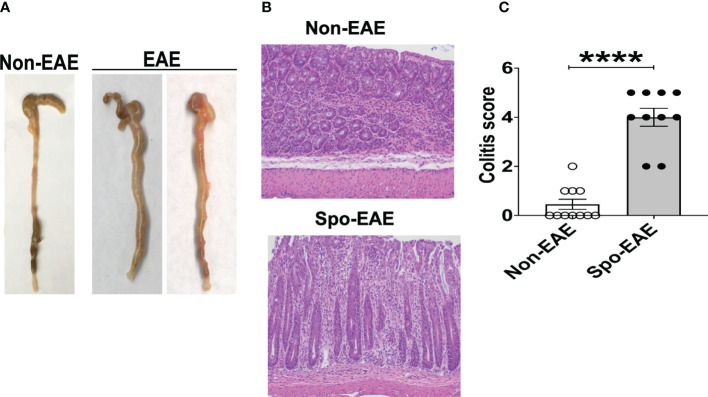
Development of colitis in spontaneous EAE mice. **(A)** Swollen colon in spontaneous EAE mice. **(B)** H&E histopathology of swollen colon isolated from spontaneous EAE and non-EAE mice. **(C)** Colitis score was evaluated by immunopathological analysis in non-EAE (n = 11) and EAE mice (n= 10) as described in materials and methods. Mean ± SEM. ****P <0.0001.

Since large intestinal neutrophil infiltration is associated with colitis (48, 49), we examined colitis development in EAE mice. The large intestinal epithelium was markedly hyperplastic, which coincided with swelling of the colon tissue, a feature of chronic colitis (**Figure 3A**). Histopathological examination yielded expanded inflammatory cells in the lamina propria of the large intestine (**Figure 3B**). Furthermore, higher colitis scores were associated with the development of EAE in 3A6/DR2a Tg mice (**Figure 3C**). In sum, these data highlight the coincident development of CNS and gut inflammation in 3A6/DR2a Tg mice. Since fecal Lcn-2 levels were increased before EAE onset (**Figure 2B**), intestinal inflammation probably preceded the onset of EAE.

### Fecal Lcn-2 levels are increased in patients with RRMS

**Figure 4:**
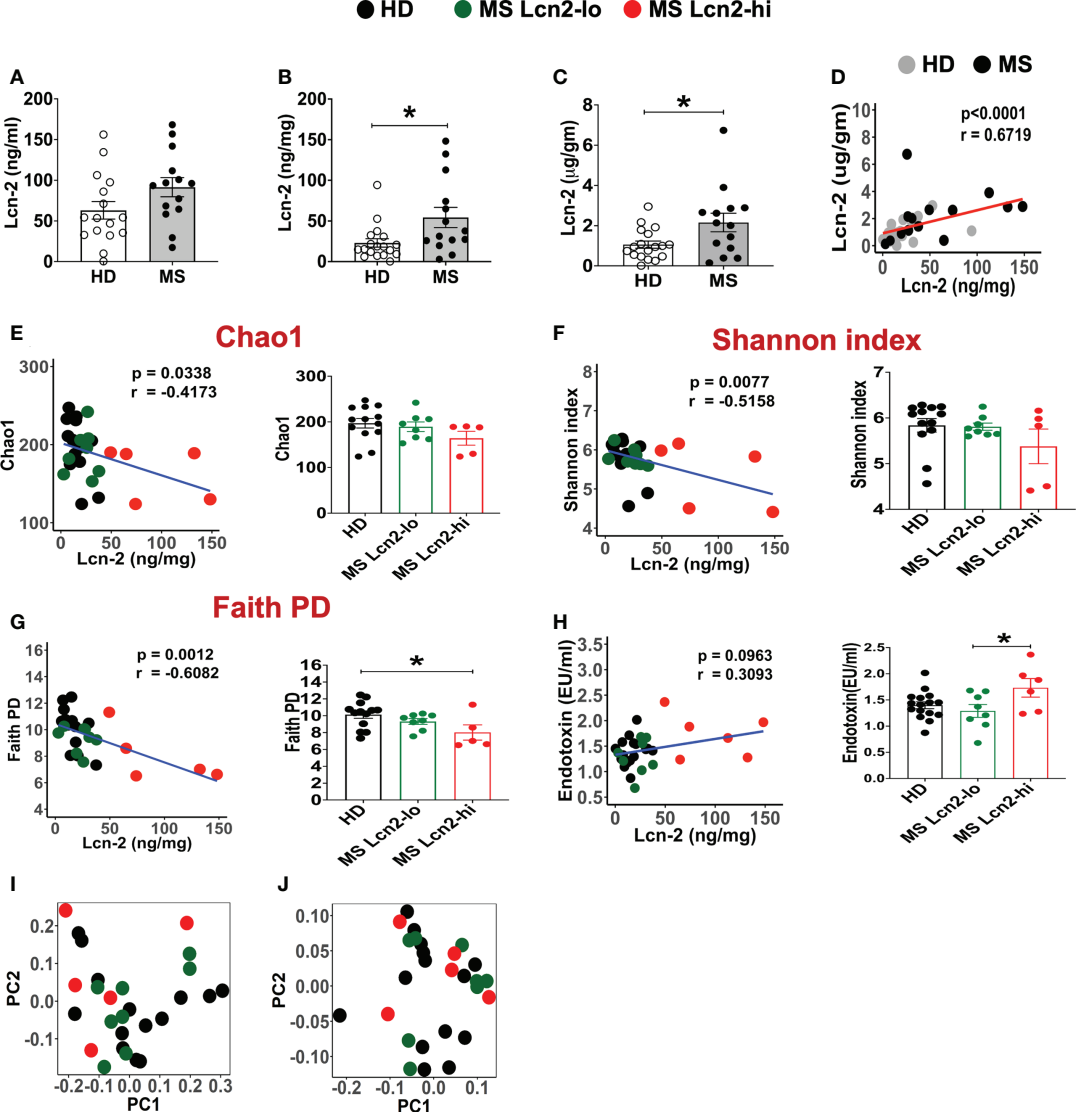
Fecal Lcn-2 is a biomarker of gut dysbiosis in RRMS patients. **(A)** Serum Lcn-2 concentration in HDs and RRMS patients. **(B–D)** Fecal Lcn-2 concentration in HDs and RRMS patients. Fecal Lcn-2 levels are shown as ng/mg total protein of fecal extract **(B)** and µg/gm fecal weight **(C)**, and fecal Lcn-2 levels between ng/mg total protein and µg/gm fecal weight are significantly correlated **(D)**. RRMS patients (n = 14) and HDs (n = 18) are shown. (E-G: left graph) Spearman correlations between fecal Lcn-2 levels and alpha diversity based on Chao1 index **(E)**, Shannon index **(F)**, and Faith phylogenetic diversity score **(G)**. (**E–G** right graph) RRMS subjects were divided into Lcn-2-low (<50 ng/mg total protein) and Lcn-2-high (>50 ng/mg total protein) groups, and alpha diversity based on Chao1 index, Shannon index, and Faith phylogenetic diversity score in HDs (n=13), MS Lcn-2-low (n=8), and MS Lcn-2-high (n=5) subjects are shown. **(H)** Spearman’s correlation between fecal Lcn-2 and serum endotoxin levels (left graph). Endotoxin levels in the serum of HD (n = 16), Lcn-2-low (n = 8) and Lcn-2-high MS subjects (n = 6) (right graph). **(I, J)** PCoA plot of beta-diversity by Unweighted UniFrac distance analysis **(I)**, and Weighted Unifrac distance analysis **(J)**. Each dot represents an individual subject. Mean ± SEM. *P < 0.05.

**Figure 5:**
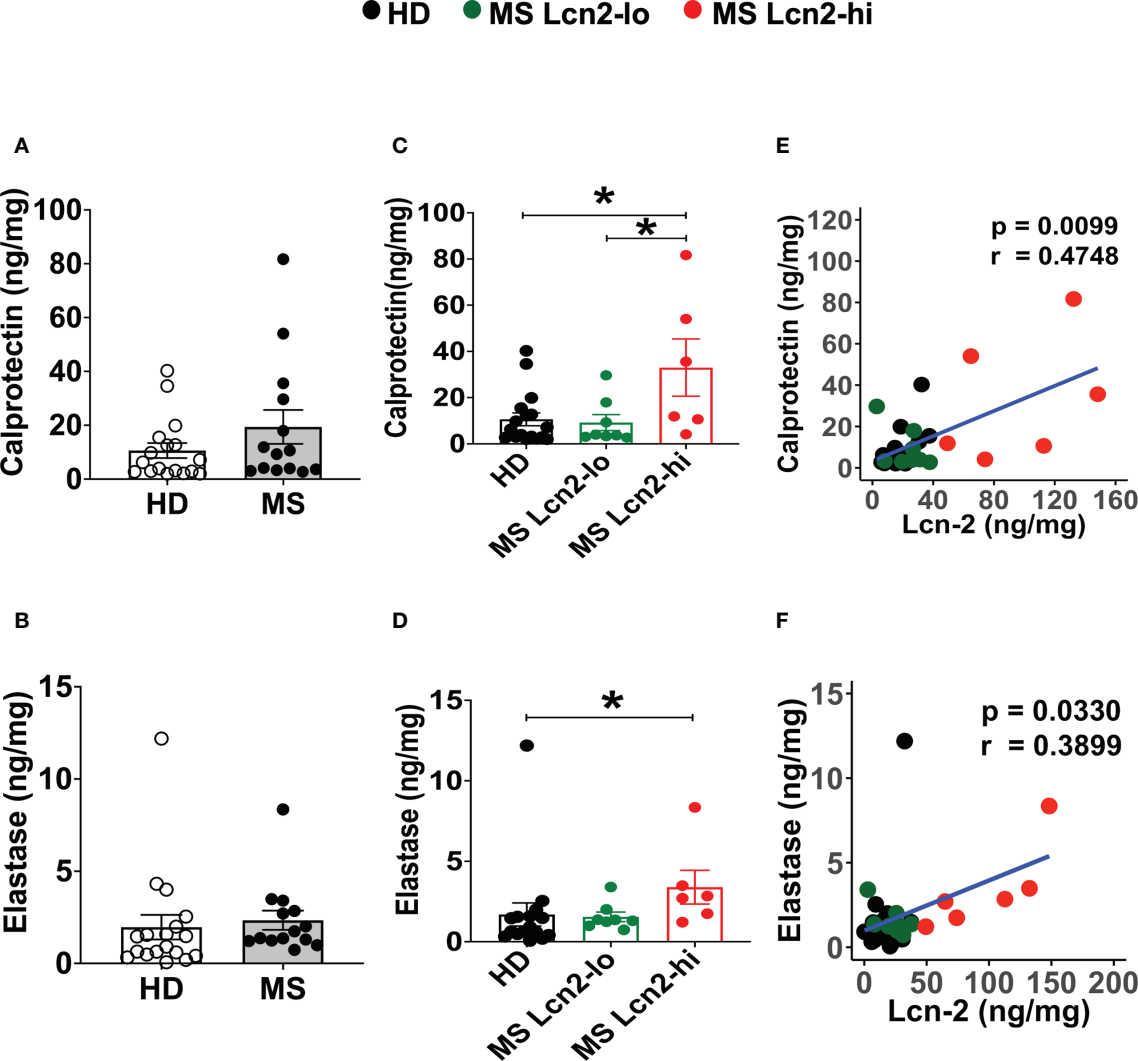
Fecal calprotectin and elastase levels in HDs and RRMS patients. **(A, B)** Fecal calprotectin levels **(A)** and fecal elastase levels **(B)** in RRMS (n = 14) and HDs (n = 18) are shown. **(C, D)** The levels of fecal calprotectin **(C)** and fecal elastase **(D)** in HDs, MS Lcn-2-low (<50 ng/mg total protein), and MS Lcn-2-high (>50 ng/mg total protein) subjects are shown. **(E, F)** Spearman correlation between fecal Lcn-2 and fecal calprotectin **(E)** or fecal elastase **(F)** is shown. HDs (n=18), MS Lcn-2-low (n=8), and MS Lcn-2-high (n=6). Mean ± SEM. *P < 0.05.

To examine the association between RRMS and gut dysbiosis, serum and fecal Lcn-2 levels of RRMS patients and healthy donors (HDs) were analyzed. No significant difference in serum Lcn-2 levels was observed between the two groups (**Figure 4A**). Cytokine secretion assays indicated that the production of pro-inflammatory cytokines, such as IFN-γ, IL-6, and IL-1β, and an anti-inflammatory cytokine, IL-10, in PBMCs was also not significantly different, except for IL-17A which was elevated in the RRMS patients compared to HDs (**Supplementary Figure 1**). On the other hand, fecal Lcn-2 levels were significantly higher in the RRMS group compared to the HD group (**Figures 4B–D**), and the increase in fecal Lcn-2 levels significantly correlated with a decrease in microbial alpha diversity (**Figures 4E–G**). Since we observed fecal Lcn-2-high (>50 ng/mg total protein) and Lcn-2-low groups in RRMS patients, we examined microbial diversity in HDs, MS Lcn-2-low, and MS Lcn-2-high groups. Alpha diversity (Faith phylogenetic diversity) was reduced in the MS Lcn-2-high group compared to the HD and MS Lcn-2-low groups (**Figure 4G** right graph), although a significant distance between MS Lcn-2-high and the MS Lcn-2-low/HD groups was not observed by UniFrac distance analysis (**Figures 4I, J**). Interestingly, serum endotoxin levels were increased in the MS Lcn-2-high group compared to the MS Lcn-2-low group (**Figure 4H** right graph), suggesting an association between intestinal inflammation and gut permeability. Since fecal calprotectin and neutrophil elastase are other biomarkers of intestinal inflammation (50, 51), we examined their levels in fecal samples. As expected, fecal calprotectin and neutrophil elastase levels were increased in the MS Lcn-2-high group compared to the MS Lcn-2-low and HD groups (**Figures 5C, D**). Furthermore, fecal calprotectin and neutrophil elastase levels correlated significantly with fecal Lcn-2 levels (**Figures 5E, F**). We also examined the correlation between alpha diversity and these two biomarkers of intestinal inflammation. As shown in **Supplementary Figure 2**, the increase in fecal Lcn-2 was mostly significantly correlated with a decrease in alpha diversity among the biomarkers of intestinal inflammation. Collectively, these data suggest that increase in fecal Lcn-2 levels is associated with gut dysbiosis in MS patients.

### Fecal Lcn-2 levels and microbial composition in RRMS

**Figure 6:**
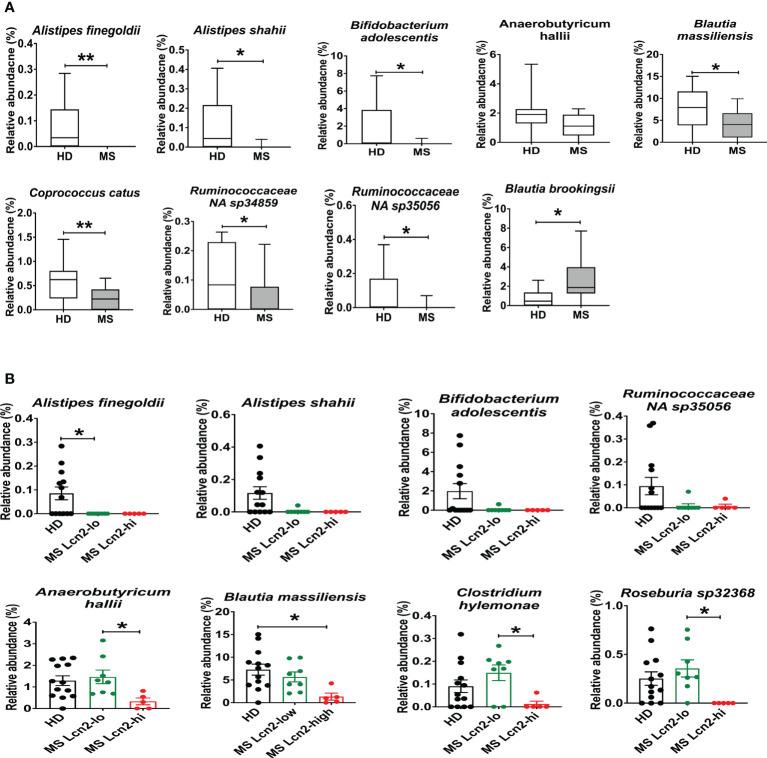
Enteric microbial composition in HDs and RRMS patients. **(A)** Enteric microbial composition in HDs and RRMS groups. The list of species exhibits changes in relative abundance of each bacterial taxa between RRMS (n=13) and HDs (n=13) groups. **(B)** Enteric microbial composition in HDs, MS Lcn-2-low and MS Lcn-2-high groups. The list of species exhibits changes in relative abundance of each bacterial taxa between HDs (n=13), MS Lcn-2-low (n=8) and MS Lcn-2-high (n=5) groups. Mean ± SEM. *P < 0.05, **P < 0.01.

To determine the association between fecal Lcn-2 levels and microbial abundance, we first evaluated microbial abundance in RRMS patients and HDs. 16S rRNA gene sequencing showed a decrease in *Alistipes finegoldii, Alistipes shahii, Bifidobacterium adolescentis*, *Anaerobutyricum* (*Eubacterium*) *hallii, Blautia massiliensis, Coprococcus catus, Ruminococcaceae NA sp 34859 and Ruminococcaceae NA sp 35056*, and an increase in *Blautia brookingsii* in RRMS compared to HDs (**Figure 6A**). Notably, *Alistipes finegoldii* and *Bifidobacterium adolescentis* have been reported as bacteria that can suppress intestinal inflammation (52, 53), and *Coprococcus catus* and *Anaerobutyricum* (*Eubacterium*) *hallii* are short-chain fatty acid (SCFA)-producing bacteria that are found reduced in IBD patients (54–56). Next, we examined which bacteria are depleted in MS Lcn-2-high patients. While *Alistipes finegoldii, Alistipes shahii*, and *Bifidobacterium adolescentis* were depleted in both MS Lcn-2-low and MS Lcn-2-high patients, we found that *Anaerobutyricum* (*Eubacterium*) *hallii, Blautia massiliensis*, *Clostridium hylemonae*, and *Roseburia sp 32368* were depleted only in MS Lcn-2-high patients (**Figure 6B** and **Supplementary Figure 3**). These data suggest that certain types of beneficial bacteria are depleted in RRMS patients and that the additional depletion of *Anaerobutyricum* (*Eubacterium*) *hallii*, *Blautia massiliensis*, *Clostridium hylemonae*, and *Roseburia sp 32368* may favor the development of intestinal inflammation in RRMS patients.

### Decrease in the relative abundance of *Blautia massiliensis* was associated with a reduction in blood SCFA levels

**Figure 7:**
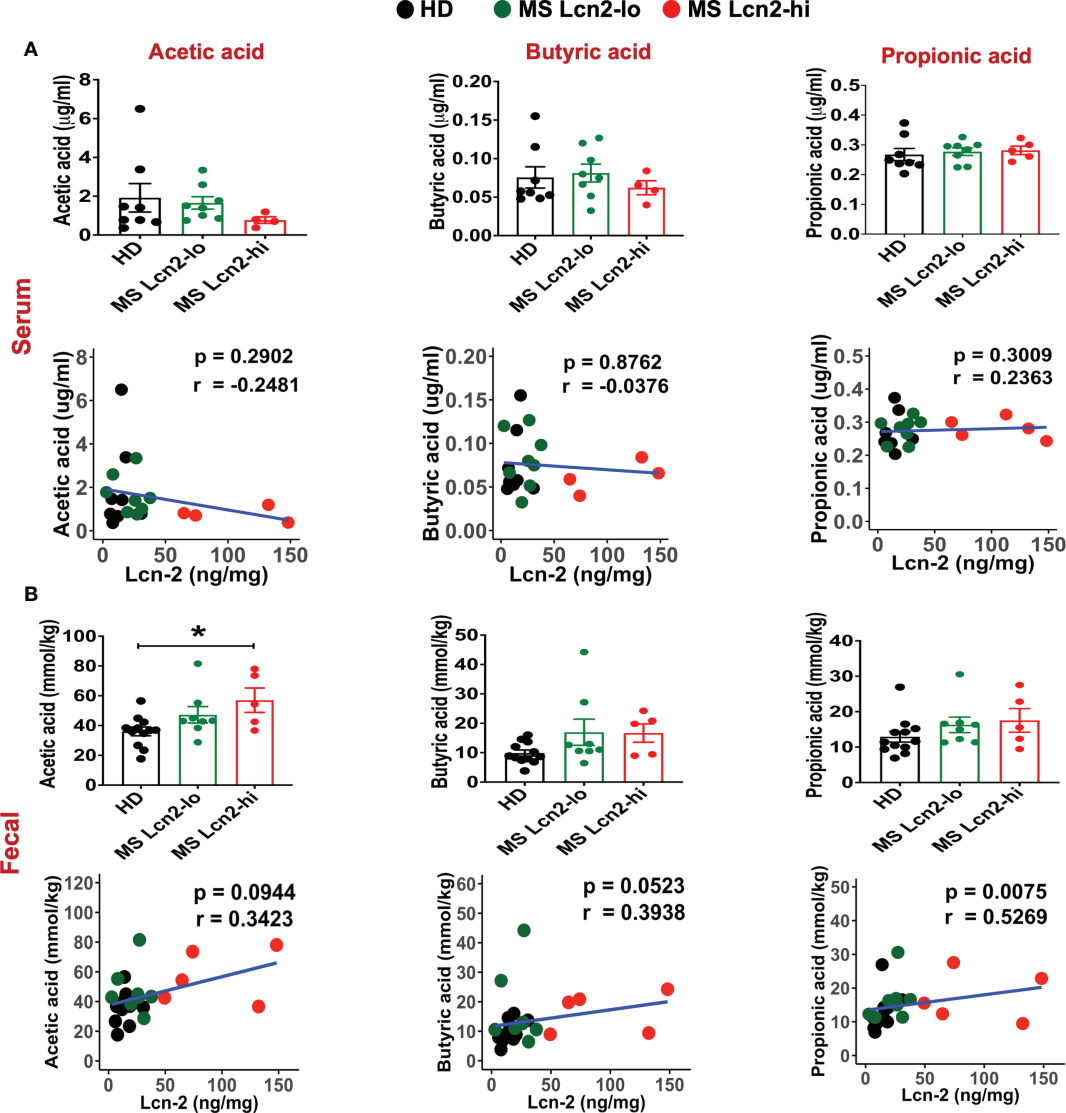
SCFAs levels in the serum and fecal samples. SCFAs levels in the serum **(A)** and fecal samples **(B)** isolated from HDs, MS Lcn-2-high, and MS Lcn-2-low groups, and correlation between SCFAs and fecal Lcn-2 levels is shown. HDs (n=8), MS Lcn-2-low (n=8), and MS Lcn-2-high (n=5). *P ≤ 0.05.

**Figure 8:**
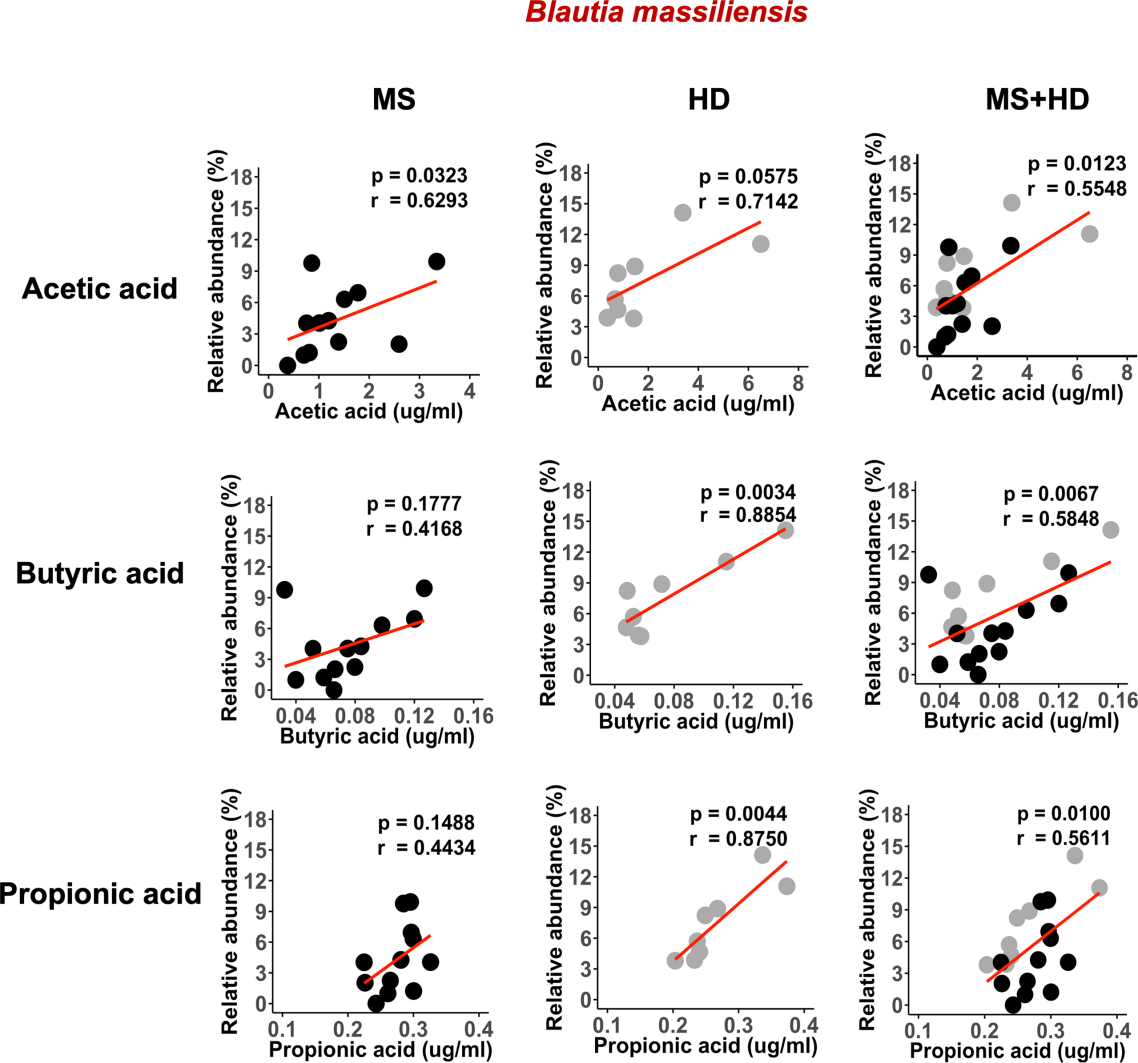
*Blautia massiliensis* is associated with serum SCFAs levels. Correlation of *Blautia massiliensis* with serum acetic acid, butyric acid, or propionic acid levels. HDs; n=8, RRMS; n=12.

A recent study showed that blood SCFA levels are directly linked to health maintenance during MS and metabolic diseases (57, 58); therefore, we examined the association between serum SCFA levels and underlying MS-linked intestinal inflammatory activity using Lcn-2 as a readout. Interestingly, we observed a decreasing trend in serum acetate levels in MS Lcn-2-high patients compared to HDs (**Figure 7A**). Since blood serum SCFA levels are determined based on the initial production of SCFAs by gut microbes and local absorption of SCFAs by the colonic epithelium, fecal SCFA levels were also examined. Interestingly, the level of fecal SCFAs was increased in the MS Lcn-2-high participants (**Figure 7B**) as observed in metabolic diseases (59, 60). Since intestinal inflammation suppresses the gene expression of SCFA transporters in the intestinal epithelium (61, 62), underlying intestinal inflammation might be actively suppressing SCFA absorption, thereby decreasing the blood circulation of SCFAs. Among MS-associated bacteria, a decrease in the relative abundance of *Blautia massiliensis* was most significantly associated with a reduction in acetic acid in the serum (**Figure 8** and **Supplementary Figure 4**). Since *Blautia massiliensis* is a highly abundant bacterium (relative abundance: 8.3 ± 4.1% in HDs versus 3.7 ± 3.4% in RRMS), its reduced abundance in MS may significantly impact the reduction in blood SCFA levels mediated by underlying intestinal inflammation.

## Discussion

In this study, we show that Lcn-2 produced by infiltrated gut neutrophils is a fecal biomarker for EAE- and MS-associated gut dysbiosis. Since peripheral T cells migrate into the gut under steady conditions (63), increases in peripheral pro-inflammatory T cells may lead to increased infiltration of cells into the gut during CNS autoimmune disease. Accordingly, we observed the infiltration and/or expansion of MBP-specific Th17 cells in the large intestine during EAE development in our animal model (**Figures 1C–E**). This is consistent with earlier studies showing that MOG-specific Th17 cells can migrate into the gut during the development of EAE (35, 36). Interestingly, the enrichment of Th17 cells in the gut was also reported in RRMS patients (2). The augmented production of IL-17A coordinates neutrophil infiltration into the gut (64), and infiltrated neutrophils can eliminate luminal microbes that translocate the epithelium and invade the mucosa. Indeed, we observed that neutrophils infiltrate the large intestines of 3A6/DR2a Tg mice that develop spontaneous EAE. In the setting of MS, similar mechanisms may be involved in the recruitment of neutrophils *via* IL-17A produced by Th17 cells.

Neutrophils mediate pathogenic microorganism removal by phagocytosis, neutrophil extracellular trap (NET) formation, and by the release of reactive oxygen and nitrogen species and antimicrobial factors, such as neutrophil elastase, Lcn-2, and calprotectin (65). Therefore, antimicrobial mediators produced by infiltrated neutrophils could be involved in the induction of gut dysbiosis. Indeed, increased levels of fecal Lcn-2, neutrophil elastase, and calprotectin levels were associated with reduced intestinal microbiota diversity in RRMS patients. Among these three mediators, fecal Lcn-2 levels were most significantly associated with MS-linked gut dysbiosis (**Supplementary Figure 2**). Neutrophils additionally participate in gut inflammation by producing high levels of *reactive oxygen species* (ROS), proteases, and pro-inflammatory factors (IL-8, TNF-α, and leukotriene B4) that damage the epithelial barrier and recruit monocytes into the gut (65). NETs released from neutrophils are also associated with inflammation and are involved in permeabilizing the gut (66–68). Furthermore, elastase released by infiltrated neutrophils has been implicated in the pathogenesis of inflammatory bowel disease (IBD) and reflects disease activity during ulcerative colitis (69–71). Therefore, the coordinated gut infiltration of Th17 cells and neutrophils may be contributing to the pathogenesis of mucosal inflammation in CNS autoimmune diseases.

MS has been reported to be associated with IBD (28–32, 72, 73), and the prevalence of IBD is increased in MS patients compared to the general population (72, 73). Increased prevalence of demyelinating diseases was also observed in IBD patients (31). This linked association suggests a bidirectional comorbidity between MS and IBD. Furthermore, genome-wide association studies involving ulcerative colitis and Crohn’s disease have identified shared risk loci between MS and IBD (34), which suggests that common pathological mechanisms affect both conditions. Our observation that 3A6/DR2a Tg mice co-develop EAE and colitis is consistent with observations made in humans (**Figure 3**). Furthermore, we previously reported that the expansion of *Bacteroides vulgatus* was significantly associated with spontaneous EAE in 3A6/DR2a Tg mice (44). *B. vulgatus* is a bacterium which localizes in the large intestine and is associated with colitis (74, 75). It is possible that the chronic infiltration of neutrophils and production of pro-inflammatory mediators induced by *B. vulgatus* may trigger colitis in 3A6/DR2a Tg mice. In addition, the expression of MBP in enteric glial cells (76, 77) and 3A6/DR2a Tg animals’ partial immunodeficiency stemming from allelic exclusion, which is caused by the expression of a transgenic TCR, may promote the development of colitogenic T cells in 3A6/DR2a Tg mice.

Since Lcn-2 is induced by pro-inflammatory cytokines in a NF-κB-dependent manner, Lcn-2 is suited to be a biomarker of inflammation (21). An increased concentration of Lcn-2 in MS patients’ plasma and cerebrospinal fluid suggests a pathological role for Lcn-2 in MS (78). The expression of Lcn-2 in microglia and astrocytes is increased in EAE mice, and disease severity is reduced in Lcn-2 null mice compared to wild-type mice (79). Lcn-2 can also promote the development of Th1 and Th17 cells (79) and M1 microglia (80). In addition, Lcn-2 can suppress remyelination (81) and induce apoptosis in neurons (82). These data suggest that Lcn-2 could play a pathological role in MS. However, the protective and pathological roles of Lcn-2 in colitis are still controversial. Lcn-2 prevents intestinal inflammation and spontaneous colitis in IL-10 null mice by enhancing phagocytic bacterial clearance in macrophages and changing microbial composition, suggesting a protective role in colitis (19, 83). In contrast, Lcn-2 deficiency can protect mice from dextran sodium sulfate and *Salmonella Typhimurium*-induced colitis, suggesting a pathogenic role of Lcn-2 in colitis (84, 85). Therefore, Lcn-2 may possess a dual function in the pathogenesis of colitis.

We examined serum and fecal Lcn-2 levels in MS patients and HDs. Although we observed an increasing trend of serum Lcn-2 levels in MS patients compared to HDs (**Figure 4A**), fecal rather than serum Lcn-2 levels were significantly associated with MS (**Figures 4B, C**). Production of pro- and anti- inflammatory cytokines in PBMCs was also not significantly different except for IL-17A (**Supplementary Figure 1**). The decreased inflammatory responses in peripheral blood could be attributed to the use of disease-modifying therapies (DMT) in most of the RRMS participants (**Table 1**). As fecal calprotectin, elastase, and Lcn-2 are well-known biomarkers of intestinal inflammation (23, 51, 86), we examined the levels of these factors in fecal samples collected from RRMS patients and HDs. Fecal Lcn-2 levels were more significantly associated with RRMS than fecal calprotectin and elastase levels (**Figures 4B, C**, **5A–D**). A subset of RRMS patients recorded a higher fecal Lcn-2 concentration ranging between 2.6-6.7 μg/gm (**Figure 4C**). This range of Lcn-2 levels indicates a mild activity of intestinal inflammation according to previous studies (22, 24). Since fecal Lcn-2 is a sensitive biomarker for intestinal inflammation compared to other biomarkers (24), MS-associated intestinal inflammation and gut dysbiosis can be detected by the fecal Lcn-2 assay. Taxonomy analysis showed that the increase in fecal Lcn-2 levels was associated with a decrease in alpha diversity among intestinal microbiota (**Figures 4E–G**). Reduced alpha diversity has previously been observed in autoimmune diseases, including MS and IBD (87, 88), and has also been associated with low-grade inflammation (89). Therefore, measuring fecal Lcn-2 levels could be helpful in monitoring MS-related gut dysbiosis and intestinal inflammation. Microbial abundance analysis also showed that *Alistipes finegoldii, Alistipes shahii*, and *Bifidobacterium adolescentis* were depleted in both Lcn-2-low and Lcn-2-high MS patients, whereas *Anaerobutyricum* (*Eubacterium*) *hallii, Blautia massiliensis*, *Clostridium hylemonae*, and *Roseburia sp 32368* were depleted only in MS Lcn-2-high patients (**Figure 6B** and **Supplementary Figure 3**). These data suggest that *Anaerobutyricum* (*Eubacterium*) *hallii, Blautia massiliensis*, *Clostridium hylemonae*, and *Roseburia sp 32368* may play a pivotal role in MS-associated intestinal inflammation.

Recent studies have proposed that blood SCFA levels are more directly linked to health maintenance than fecal SCFA levels (57). A decrease in SCFA levels is also associated with an increase in pathogenic immune cells in MS patients (58). Therefore, we examined the effect of gut dysbiosis-mediated intestinal inflammation (fecal Lcn2 levels) on SCFA levels in the blood and feces. Since intestinal inflammation suppresses the expression of SCFA-transporter expressed on the intestinal epithelium (61, 62), intestinal inflammation could suppress the absorption of SCFA and decrease SCFA levels in the blood. We observed a decreasing trend in acetate and butyrate in the blood, while an increasing trend in acetate, butyrate, and propionate in the fecal samples of Lcn-2 high MS patients (**Figures 7A, B**). Since more than 95% of SCFA produced by intestinal microbiota are absorbed in the gut, the decrease in SCFA absorption by dysbiosis-mediated intestinal inflammation may lead to an increase in SCFA levels in fecal samples. Indeed, recent studies have also shown that increased fecal SCFA levels are associated with gut dysbiosis in metabolic diseases (59, 60). It is worth pointing out that previous studies showed lower fecal SCFA levels in fecal samples collected from MS patients compared to HDs (3, 90). The contrast with our study could be due to differences in dietary habits and genetic background that may affect the growth of SCFA-producing bacteria and expression of the SCFA transporter. A recent study also indicated that fecal and blood SCFA levels in MS patients vary by the disease modifying therapies used (91). Therefore, several factors could contribute to the variations in levels of SCFA in the feces and blood among different studies. We also examined intestinal bacteria that are associated with a decrease in serum SCFA levels and found that depletion of *Blautia massiliensis* was significantly associated with a reduction in serum levels of acetic acid, butyric acid and propionic acid (**Figure 8** and **Supplementary Figure 4**). *Blautia massiliensis* is the third most abundant intestinal bacteria in the participants. Since *Blautia massiliensis* is a newly identified strain (92) and its role in human health is not well understood, further studies are required to explore the involvement of *Blautia massiliensis* in MS-mediated intestinal inflammation/gut dysbiosis and disease activation.

In summary, we demonstrated in a humanized spontaneous EAE model of MS that CNS and gut inflammation can coexist and that they are mechanistically associated with gut-infiltration of pathogenic T cells and recruitment of Lcn-2^+^ neutrophils. The observations we describe in MS patients support the findings in EAE and indicate that fecal Lcn-2 is a sensitive biological marker for gut dysbiosis in MS. It is often difficult to characterize gut dysbiosis and identify the specific bacteria linked to MS due to a heterogenous microbiome caused by genetic factors, dietary habits, and/or environmental exposures*. Therefore*, analysis of fecal Lcn-2 levels together with taxonomic evaluation could be a useful method to examine intestinal microbial homeostasis in MS. In the future, it is important to explore the association between fecal Lcn-2 levels and clinical parameters including MS relapse rate and disease progression in a larger sample size prospective study.

## Data availability statement

The data presented in the study are deposited in the NCBI repository, accession number: PRJNA875026; https://www.ncbi.nlm.nih.gov/bioproject/PRJNA875026.

## Ethics statement

The studies involving human participants were reviewed and approved by IRB Rutgers University. The patients/participants provided their written informed consent to participate in this study. The animal study was reviewed and approved by IACUC Rutgers University.

## Author contributions

SY, NI, JM, HK, MY, RK, and SS preformed experiments and analyzed the data. SD-J, KB, and YR coordinated the collection of samples from RRMS patients and healthy donors. SY, SD-J, and KI designed research studies. KI and SD-J supervised the research and interpreted the data. SY and KI wrote the manuscript. SD-J and JM reviewed and edited the manuscript. All authors reviewed the manuscript and approved the final version.

## Funding

This study was supported by the National Multiple Sclerosis Society Research grant RG-1901-33077 (to KI and SD-J), NIH R21AI130585 (to KI and SD-J) and the Ruth Dunietz Kushner and Michael Jay Serwitz endowed Chair in Multiple Sclerosis to SD-J.

## Conflict of interest

The authors declare that the research was conducted in the absence of any commercial or financial relationships that could be construed as a potential conflict of interest.

## Publisher’s note

All claims expressed in this article are solely those of the authors and do not necessarily represent those of their affiliated organizations, or those of the publisher, the editors and the reviewers. Any product that may be evaluated in this article, or claim that may be made by its manufacturer, is not guaranteed or endorsed by the publisher.
