# Supplementary material for: Fecal Lcn-2 level is a sensitive biological indicator for gut dysbiosis and intestinal inflammation in multiple sclerosis
Source: Front Immunol. 2022 Oct 21;13:1015372. doi: 10.3389/fimmu.2022.1015372 (PMC9634083; doi:10.3389/fimmu.2022.1015372)
Supplement: Supplementary file 1 [file DataSheet_1.pdf]

Supplementary Material

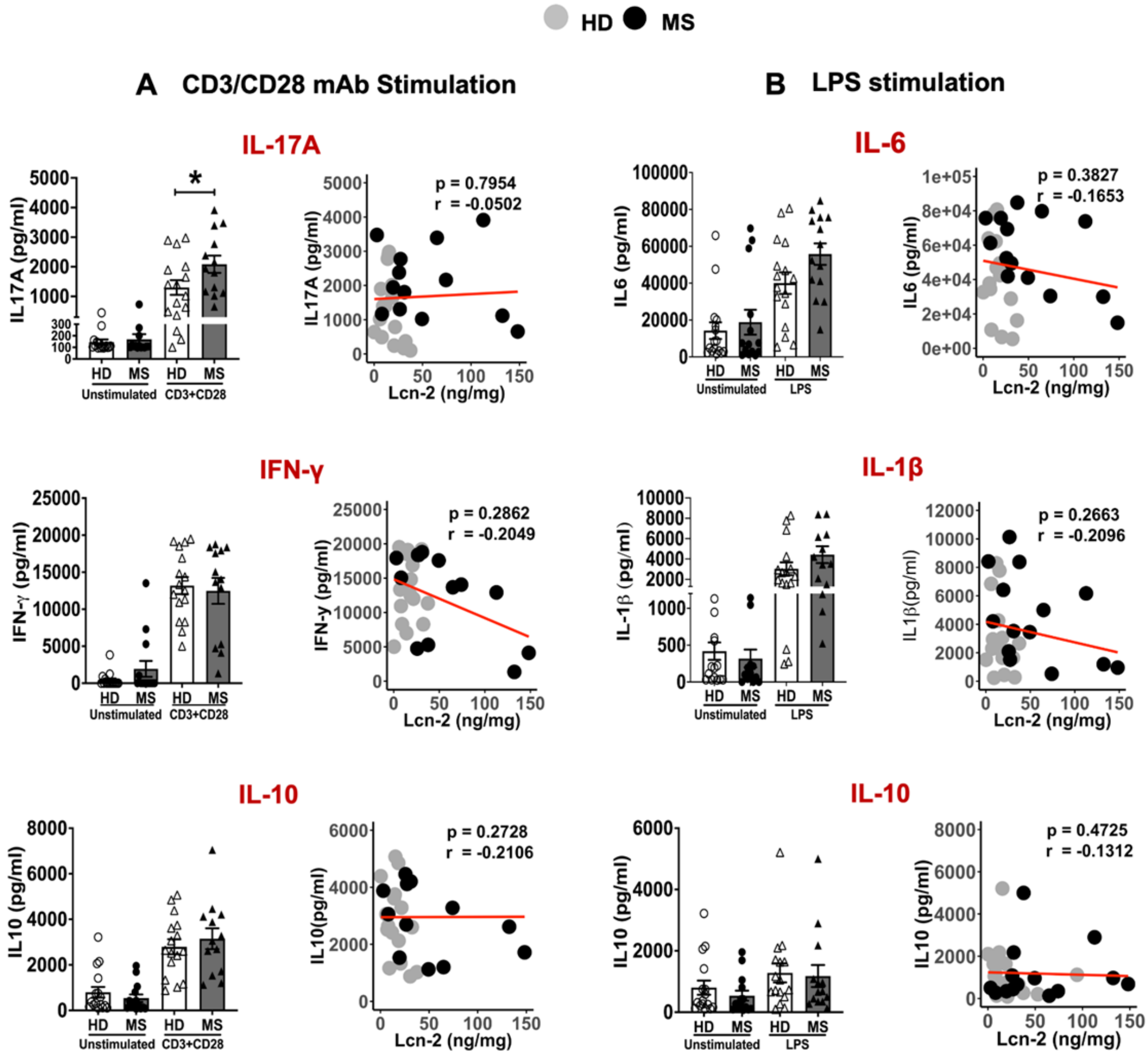

Supplementary Figure 1. Cytokine production by PBMC in HDs and RRMS patients.

(A) Production of IL17A, IFN-γ, and IL10 in PBMCs isolated from HDs and RRMS patients with/without CD3/CD28 Abs stimulation, and their correlation with fecal Lcn-2 levels. (B) Production of IL6, IL1β and IL10 in PBMC isolated from HDs and RRMS patients with/without LPS stimulation, and their correlation with fecal Lcn-2 levels. Mean ± SEM. *P < 0.05.

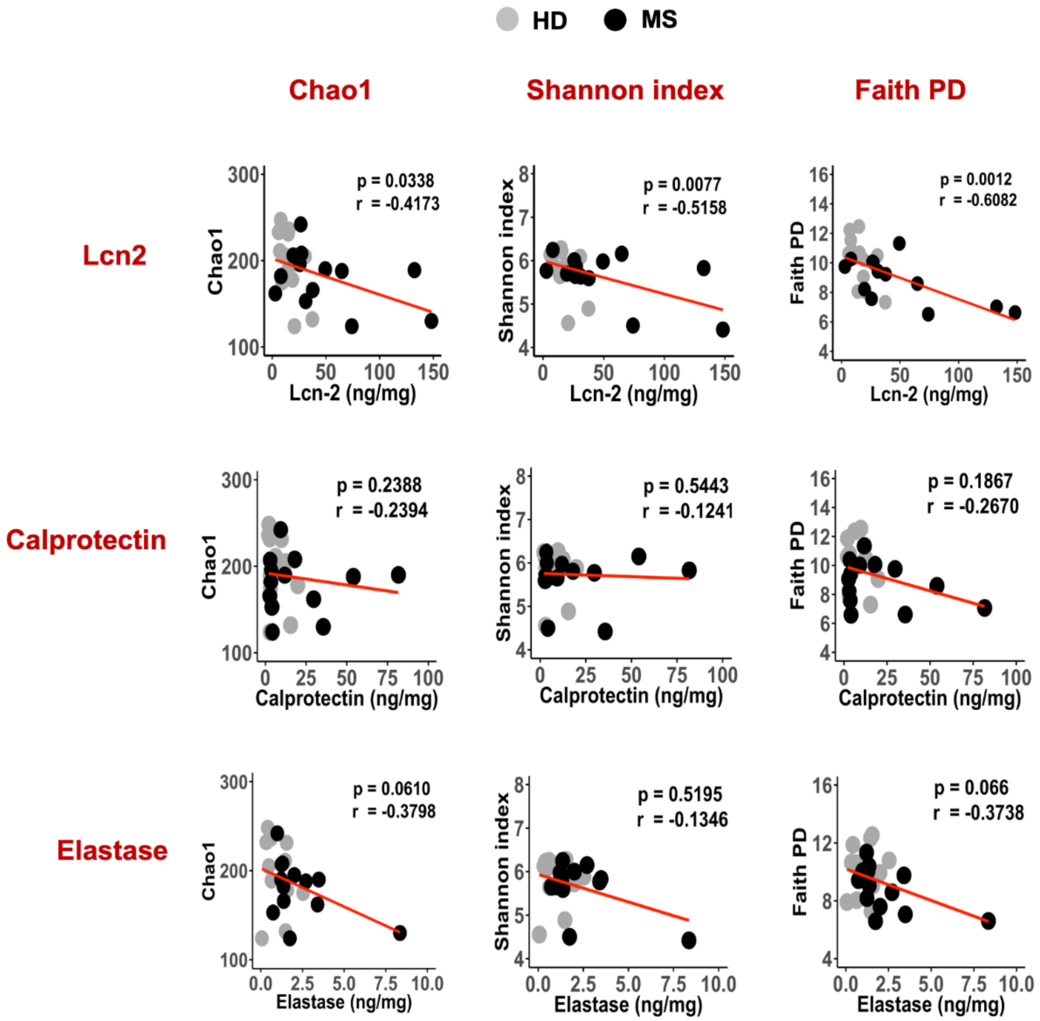

Supplementary Figure 2. Correlation between gut inflammation markers and alpha diversity.

Spearman correlations between alpha diversity (Chao1, Shannon index, and Fath PD) and Lcn-2, calprotectin, and elastase are shown. Each dot represents an individual subject. HDs; n=13, RRMS; n=13.

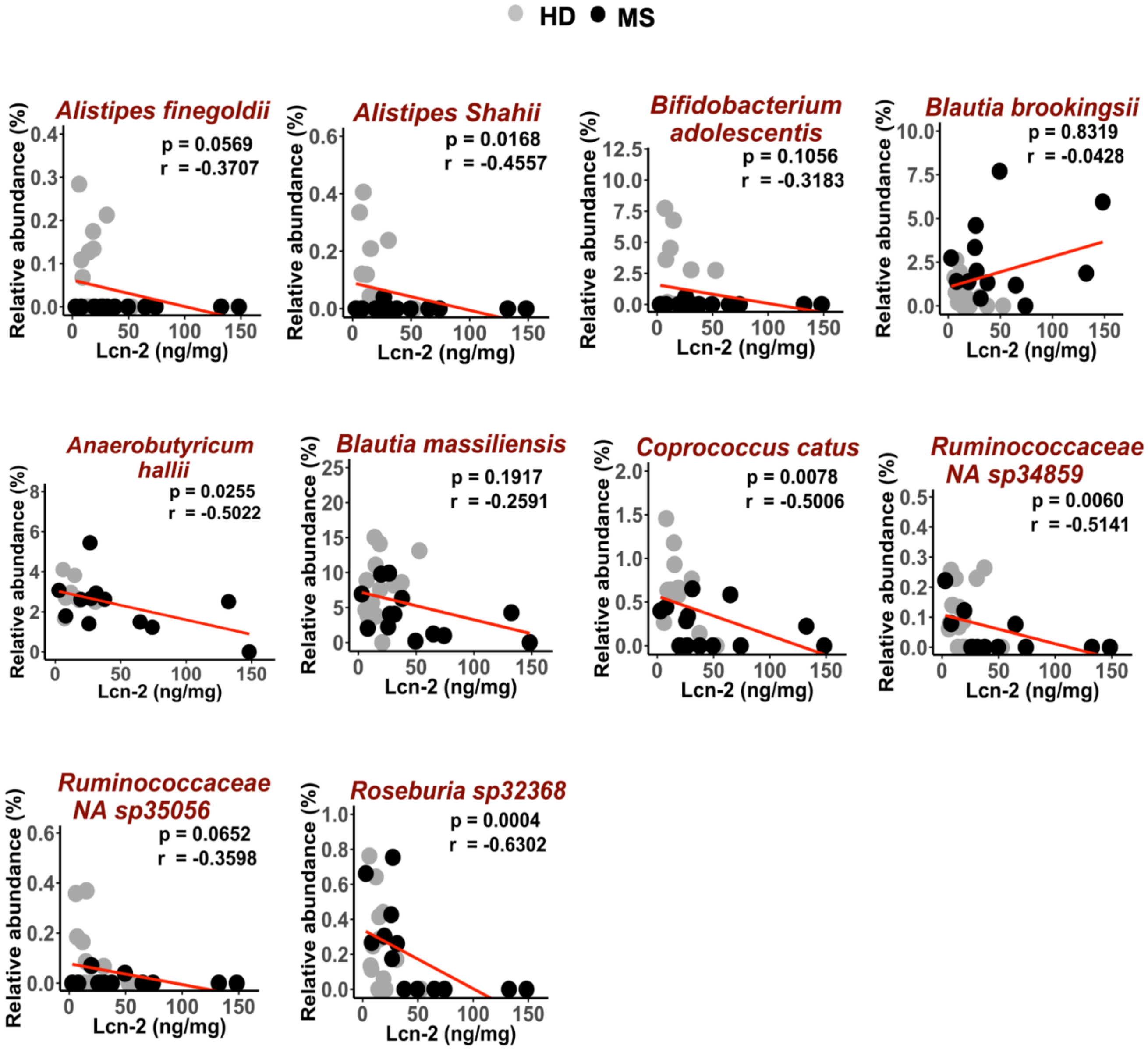

Supplementary Figure 3. Correlation between fecal Lcn-2 levels and RRMS-associated enteric bacteria. Spearman correlation between fecal Lcn-2 and bacterial taxa; *Alistipes finegoldii*, *Alistipes shahii*, *Bifidobacterium adolescentis*, *Blautia brookingsii*, *Anaerobutyricum* (*Eubacterium*) *hallii*, *Blautia massiliensis*, *Coprococcus catus*, *Ruminococcaceae NA sp 34859*, *Ruminococcaceae NA sp 35056, and Roseburia sp32368.* Each dot represents an individual subject.

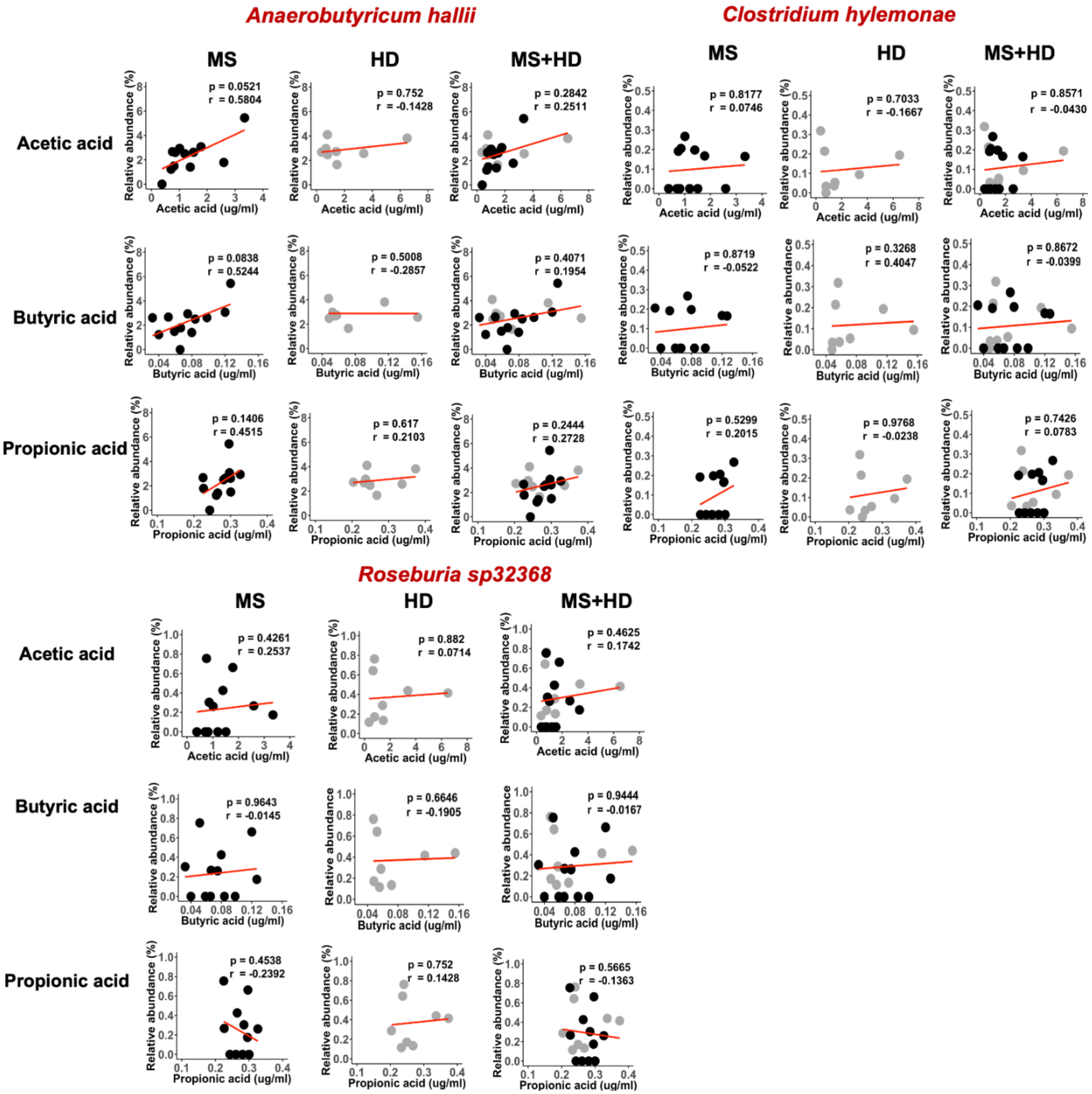

Supplementary Figure 4. Association of MS-associated bacteria with serum SCFAs levels.

Correlation of *Anaerobutyricum* (*Eubacterium*) *hallii*, *Clostridium hylemonae, and Roseburia sp 32368* with serum acetic acid, butyric acid, or propionic acid levels. HDs; n=8, RRMS; n=12.
